# DIMPSAR: Dataset for Indian medicinal plant species analysis and recognition

**DOI:** 10.1016/j.dib.2023.109388

**Published:** 2023-07-14

**Authors:** Pushpa B R, N. Shobha Rani

**Affiliations:** Department of Computer Science, School of Computing, Mysuru, Amrita Vishwa Vidyapeetham, India

**Keywords:** Plant classification, Leaf images, Whole plant images, Mobile captured images, Plant/leaf analysis

## Abstract

Mobile-captured images of medicinal plants are widely used in various research investigations. Machine vision-based tasks such as the identification of plant species types for intelligent imaging device applications take a significant part in it. Botanists, farmers and researchers can reliably identify medicinal plants with the help of images captured using smartphones.  Mobile captured images can be used for quality control to make sure that the right plant species are being used in pharmaceutical products. In the field of education, pictures of medicinal plants and their usage can be used to educate learners, medical professionals, and the general public. Further, various research investigations in the area of chemistry, pharmacology, the therapeutic potential of medicinal plants, images can be employed.

In this paper, we contribute a dataset of Indian medicinal plant species. The dataset is collected from different regions of Karnataka and Kerala. Datasets include characteristics such as multiple resolutions, varying illuminations, varying backgrounds, and seasons in the year. The datasets consist of 5900 images of forty plant species and single leaf images of eighty plant species consisting of 6900 samples obtained from real-time conditions using smartphones. The datasets contributed would be useful to researchers to investigate on development of algorithmic models based on image processing, machine learning, and deep learning concepts to educate about medicinal plants. The dataset can be accessed by anybody, without charge, at DOI:10.17632/748f8jkphb.2, 10.17632/748f8jkphb.3

Specifications table

**Table:** 

Subject	Computer Science; Agricultural and Biological Sciences

Specific subject area	Image processing, Computer vision and Pattern recognition; Plant species recognition
Type of data	Images
How the data were acquired	In the proposed work, there is no fixed setup being followed to acquire the images, realistic image-capturing context for smartphone image acquisition is considered while taking photos of plants. A group of ten smartphone users is assigned to capture the dataset. Images are acquired using different smartphones with a camera resolution of Nokia N95(5MP), Xiaomi Redmi 1S(8MP), Samsung Galaxy S9(12MP), Xiaomi Mi 4i(13MP) and Xiaomi Redmi Note3(16MP). Images are photographed in different weather conditions on cloudy, sunny, and windy days with varying distances, lighting, and backgrounds in multiple projection angles covering both sides of leaf patterns.
Data format	Raw
Description of data collection	Images collected as part of dataset collection include real-time challenges related to the recognition and identification process of plants. All species considered are medicinal and captured with fixed lens smartphone cameras with different perspectives of projection angles, color variations of leaf, multiple resolutions and varying distances. The spatial resolution of images captured spans from 2,560×1,920 to 5312×2988. There exists an imbalanced number of samples per class that are addressed through image augmentation methods.
Data source location	Various botanical gardens are visited in and around Karnataka and Kerala to carry out dataset collection. Some of them include., 1. Medicinal plant garden Chandravana, Government Ayurveda Medical College and Hospital, Mysuru. https://goo.gl/maps/r8p66iJK2hjaLxrcA 2. Green Atmosphere plant nursery, Hebbal, Mysuru https://goo.gl/maps/4mAg8355RRniqDAr8 3. Bhudevi Farm, Jayalakshmipuram, Mysuru https://goo.gl/maps/2zDJLZPq6HYLonxj6">https://goo.gl/maps/2zDJLZPq6HYLonxj6 4. Uppala medicinal botanical garden, Kasaragod, Kerala 5. Medicinal botanical garden, Kasaragod, Kerala. Country: India https://goo.gl/maps/VA8uyK46qcsEG7LXA
Data accessibility	Repository name: Mendeley data DOI: 10.17632/748f8jkphb.3 URL:https://data.mendeley.com/datasets/748f8jkphb
Related research article	Pushpa, B. R., & Rani, N. S. (2023). Ayur-PlantNet: An unbiased light weight deep convolutional neural network for Indian Ayurvedic plant species classification. Journal of Applied Research on Medicinal and Aromatic Plants, 100459. (https://doi.org/10.1016/j.jarmap.2023.100459)

## Value of the Data

• Datasets of medicinal plants are helpful in scientific investigations in various fields for research. Exploration of the appearance and features of different plant species is useful in vision-based plant identification, phytochemical analysis, and conservation efforts.
• Datasets can be used for the development of algorithmic models based on deep/machine learning/image analysis and pattern recognition.
• Furthermore, in the area of object detection from images, the raw images can be helpful in proposing the models to address various challenges related to medicinal plant species classification, leaf region segmentation, pre-processing, shadow removal, estimate leaf count, modeling of relation to compute similarity from one plant species to another belonging to the same class and different classes and occluded leaf recognition etc.
• Datasets can be utilized to create an application that can educate the students and spread awareness on Indian medicinal plants and its health benefits to mankind.
• The images collected can be analyzed using various image processing tools to extract useful information such as plant morphology, color, and texture. This data can be used for different applications such as machine learning algorithms for the diagnosis of plant diseases.
• Images collected can help in creation of a comprehensive database comprising information related to diseases, usefulness, pests/fertilizers to be applied to prevent diseases and other industrial uses.

## 1 Objective

Ayurveda is one of the ancient medicinal systems practiced in India for several thousands of years to treat various diseases with lower cost and undesirable side effects compared to allopathy medicine. Every organ of medicinal plants such as root, leaf, stem, fruit, and seed are composed of medicinal properties. The primary objective of creating an image dataset of Indian medicinal plant species is to promote the use of ayurvedic medicinal practices and spread the knowledge related to common medicinal plants that are present around us. The awareness would help in encouraging researchers, educators, and practitioners in the field of medicinal plants to address new challenges. Also, this would lead to an increased harvest of medicinal plants that leads to the adoption of the best health practices by common people and reducing health-related risks. Specifically, the creation of the datasets can serve benefits such as plant species identification in machine/ deep learning, biodiversity conservation, medicinal plant research [1], phytochemical analysis, ayurvedic medicine, education and outreach, conservation and sustainable use.

Overall, the creation of an Indian medicinal plant dataset species can contribute to the development of innovative and sustainable approaches to their use and conservation.

## 2 Data Description

This work is exclusive as there is no standard plant organ image dataset for Indian medicinal plants in the literature as presented in the Table 1. The image acquisition process is set free from various constraining factors that usually occur in the conventional image acquisition model [2]. The images of plants are acquired invariant to season, time, lighting conditions and background making it useful for real-time plant analysis related investigations. The list of Indian medicinal plant species is contributed by intuitive exploration of medicinal values with the help of botanists, phytochemists and data sources from the Botanical survey of India. Table 1 highlights the literature worked on Indian medicinal plants using self-built datasets and Table 2 details the list of the Indian medicinal plant species that contributed as part of this work [3].

**Table 1:** Highlights of literature work on Indian medicinal plant dataset

Reference	Dataset	No. of classes	No. of samples	Attributes
[4]	Self-built	10	1300	High resolution, uniform illumination, plain background
[5]	Self-built	18	300	Uniform illumination, plain background
[6]	Self-built	50	1500	Plain background
[7]	Self-built	4	-	Plain background
[2]	Self-built	40	2515	Plain background
[8]	Mendeley dataset	30	1835	High resolution images

**Table 2:** List of medicinal plant species

Sl No	Common Name	Botanical Name	No. of samples	Sl No	Common Name	Botanical Name	No. of samples
1	Alovera	Aloe barbadensis miller	118	41	Nithya Pushpa	Catharanthus roseus	134
2	Amla	Phyllanthus emblica	67	42	Onion	Allium cepa	92
3	Noni	Morinda citrifolia	72	43	Spinach	Spinacia oleracea	149
4	Giloy (Amrutaballi)	Tinospora cordifolia	91	44	Neem	Azadirachta indica	132
5	Peepal(Arali)	Ficus religiosa	89	45	Seethashoka		47
6	Asthma weed	Euphorbia hirta	82	46	Indian blackberry (Nerale)	Syzygium cumini	62
7	Bamboo	Bambusoideae	118	47	Papaya	Carica papaya	135
8	Betel	Piper betle	114	48	Parijatha	Nyctanthes arbor- tristis	66
9	Brahmi	Bacopa monnieri	104	49	Pepper	Piper nigrum	18
10	Bringaraj	Eclipta prostrate	73	50	Pomegranate	Punica granatum	75
11	Camphor	Cinnamomum camphora	66	51	Raddish	Raphanus sativus	40
12	Castor	Ricinus communis	129	52	Rose	Rosa	106
13	Citron lime (herelikai)	Citrus medica	99	53	Coriander	Coriandrum sativum	115
14	Coffee	Coffea	83	54	Indian Borge (Doddapatre)	Coleus amboinicus	142
15	Curry leaf	Murraya koenigii	168	55	Drumstick	Moringa oleifera	56
16	Insulin plant	Costus igneus	89	56	Aak	Calotropis gigantea	81
17	Jackfruit	Artocarpus heterophyllus	110	57	Black nightshade	Solanum nigrum	63
18	Jasmine	Jasminum	49	58	Eucalyptus	Eucalyptus globulus	80
19	Ginger	Zingiber officinale	82	59	Lemon	Citrus limon	123
20	Guava	Psidium guajava	128	60	Lemon grass	Cymbopogon citratus	18
21	Henna	Lawsonia inermis	80	61	Kamakasturi	Ocimum basilicum	67
22	Hibiscus	Hibiscus rosa-sinensis	118	62	Fluted pumpkin leaf	Cucurbita moschata	92
23	Taro	Colocasia esculenta	69	63	Ganigale	Calotropis gigantea	75
24	Malabar spinach	Basella alba	79	64	Tecoma	Tecoma stans	69
25	Mango	Mangifera indica	103	65	Nagadali	Ruta graveolens	67
26	Marigold	Tagetes erecta	93	66	Tamarind	Tamarindus indica	156
27	Beans	Phaseolus	97	67	Seethapala	Annona reticulata	114
28	Chilly	Capsicum frutescens	69	68	Sapota	Manilkara zapota	45
29	Honge	Millettia pinnata	113	69	Gasagase	Papaver somniferum	79
30	Flameleaf	Ixora coccinea	76	70	Tomato	Solanum lycopersicum	62
31	Thumbe	Leucas aspera	74	71	Sampige	Magnolia champaca	61
32	Tulsi	Ocimum tenuiflorum	177	72	Globe amarnath,	Gomphrena globosa,	81
33	Turmeric	Curcuma longa	39	73	Caricature	graptophyllum_pictum	76
34	Malabar Nut	Adathoda vasica)	51	74	Spinach	Spinacia oleracea	54
35	Balloon Vine	Cardiospermum halicacabum	61	75	Butterfly pea	Clitoria ternatea	60
36	Badipala		76	76	Kasaambruga		48
37	Chakte		68	77	Nelavambu	Andrographis paniculata	90
38	Padri	Radermachera xylocarpa	73	78	Lantana	Lantana camara	76
39	Pea	Pisum	47	79	Kohlrabi	Brassica oleracea Gongylodes	73
40	Ashoka	Saraca asoca	81	80	Mint	Mentha	135

Two datasets consisting of single leaf and whole-plant species of medicinal plants are collected. Dataset of whole plant images consists of forty plant species of 5200 raw image samples and post-augmentation of 5900 image samples [9]. The leaf image dataset of 80 plant species of 6900 image samples. Table 3 gives the summary of the datasets.

**Table 3:** Detailed description of datasets collected

Type	Number of plants Species	Total images	Type of background
Medicinal leaf dataset	80	6900	Plain / Non varying intensity levels
Medicinal plant dataset	40	5900	Varying background

## 3 Experimental Design, Materials, and Methods

### 3.1 Data collection

The authors collected the dataset from various botanical gardens in and around Mysuru, Karnataka and Kasaragod, Kerala, India.

1. Medicinal plant garden Chandravana, Government Ayurveda Medical College and Hospital, Mysuru.
2. Green atmosphere, plant nursery, Hebbal, Mysuru
3. Bhudevi Farm, Jayalakshmipuram, Mysuru
4. Uppala medicinal botanical garden, Kasaragod, Kerala
5. Medicinal botanical garden, Kasaragod, Kerala

The Chandravana medicinal garden consists of plant species that are rich in medicinal values, rare and endangered species are preserved by providing a suitable environment. The garden consists of more than three hundred plant varieties. The other medicinal plant gardens have medicinal plant species that are commonly found.

### 3.2 Data acquisition

#### 3.2.1 Medicinal leaf dataset

**Fig. 1:**
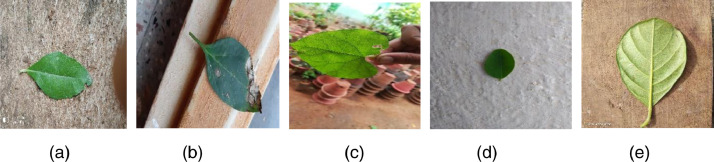
Image samples captured in the varying background (a) Leaf- Shadow (b) Leaf- Occluded (c) Leaf- Varying background (d) Leaf- Light Illumination (e) Leaf- Bright sunlight.

The dataset is created by first plucking more than 80 leaves in each species type. Initially, the leaves are cleaned to remove the dust or other particles that are present on the leaf. The leaf images are captured both in indoor and outdoor environments with natural lightning conditions and others [10]. The leaf images are placed on various background surfaces with moderate image stabilization, zoom for tiny leaves, autofocus method for standard leaf sizes. The sample of leaf images is presented in Fig. 1.

The images captured in this dataset consist of image samples that include-

a. Occluded images: The leaf images that are broken or the leaf region is not complete and partially crawled.
b. Indoor images: The images captured inside the closed environment under the natural light that are positioned at different angles with various backgrounds.
c. Outdoor images: The images are captured outside using natural lights that are placed on various backgrounds like sand, stones, green background and other objects.
d. Shadow images: The images were captured in different lighting conditions such as bright sunlight or occlusions that would result in shadow formation.
e. Leaf images with inter-class similarity where plants belong to different classes but look similar and plant species that belong to the same class and vary in their shape, texture and color.

#### 3.2.2 Medicinal plant dataset

**Fig. 2:**
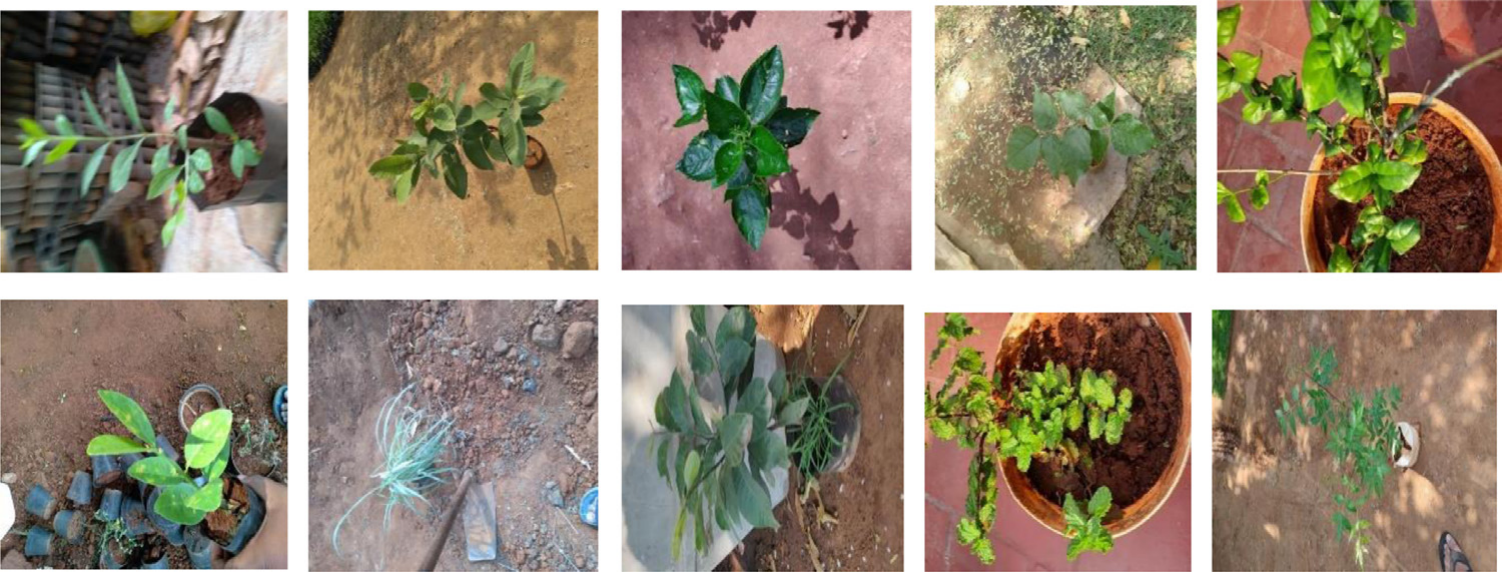
Samples of whole plant images captured in varying background

The dataset is created for forty plant species types that consist of more than 100 image samples in each species type. The images are captured using multiple mobile phones with different resolutions and other specifications [11]. The created datasets can be utilized to address the real-time plant species recognition and analysis challenges. Fig. 2 shows the sample images of datasets collected under plant datasets acquired under various conditions comprising various image acquiring factors.

### 3.3 Data nomenclature

Two dataset contributions are the medicinal leaf dataset and the medicinal plant dataset. Both datasets are captured using smartphone cameras with various real-time conditions as mentioned. Both image datasets are captured with different illuminations, occluded leaves, shadows, and varying backgrounds. The medicinal plant dataset is captured directly from farms with the specified image acquisition conditions using 5MP, 12MP, 8MP,13MP, and 16MP resolutions. The collected images are compiled into separate folders with the folder name of plant species in both medicinal leaf and plant datasets.

### 3.4 Data augmentation

**Fig. 3:**
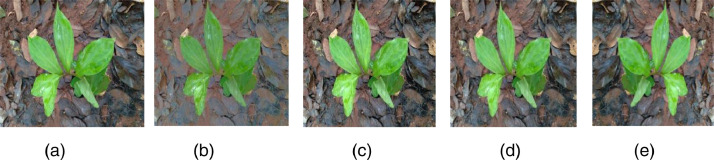
Augmented samples geometrical intensity transformations (a) Original image (b) Low contrast images (c) High contrast images (d)Rotated images (e) Flipped images.

Data augmentation is applied only on medicinal plant datasets. The medicinal plant dataset with image samples of 5200 captured with a natural background was subjected to data augmentation to reduce the dataset imbalance and cover all real-time challenges. Geometrical and intensity transformations such as image rotation is achieved by rotating the image by 180-degree, low contrast by multiplying the intensity factor by 0.6, high contrast with an intensity factor 1.5, and flip are employed by flipping the raw image in the direction to create an augmented dataset. After the geometric transformation operations, all samples are resized to fixed spatial dimensions of 600 * 450 pixels. The post-augmented dataset contains 5900 image samples and each class consists of 140-150 samples of balanced data. Fig. 3 shows the augmented image samples of medicinal plant datasets.

## Ethics Statements

This work does not involve studies with animals and humans.

## CRediT authorship contribution statement

**Pushpa B R:** Conceptualization, Formal analysis, Investigation, Data curation, Methodology, Resources, Writing – review & editing, Writing – original draft, Validation. **N. Shobha Rani:** Conceptualization, Formal analysis, Investigation, Data curation, Methodology, Resources, Writing – review & editing, Writing – original draft, Validation, Supervision.

## Declaration of Competing Interest

The authors declare that they have no known competing financial interests or personal relationships which have or could be perceived to have influenced the work reported in this article.

## Data Availability

Indian Medicinal Leaves Image Datasets (Original data) (Mendeley Data)
